# Comparative evaluation of coronally advanced flap with and without Biomesh^®^ membrane for the treatment of localized gingival recession defects – a clinical study

**DOI:** 10.25122/jml-2021-0109

**Published:** 2022-05

**Authors:** Akansha Sharma, Amit Wadhawan

**Affiliations:** 1. Department of Periodontology and Oral Implantology, Kalka Dental College and Hospital, Meerut (UP), India; 2. Department of Periodontology and Oral Implantology, Shree Bankey Bihari Dental College and Hospital, Masuri, Ghaziabad (UP), India

**Keywords:** coronally advanced flap, split-thickness flap, guided tissue regenerative membrane, periosteum

## Abstract

Numerous surgical procedures are used to correct gingival recession, like free gingival graft, pedicle graft, and connective tissue graft. Our study aimed to compare and clinically evaluate root coverage using a coronally advanced flap (CAF) with and without Biomesh^®^ membrane to treat recession type 1 (RT_1_) and type 2 (RT_2_) defects. A total of 20 systemically stable patients, both males and females between the ages of 20 and 40, with bilateral recession defects in maxillary canines and premolars, were included in the study. Patients were divided into two groups: the control group: coronally advanced flap only and the test group: coronally advanced flap with Biomesh^®^ membrane. All clinical parameters showed significant reductions from baseline, 1 month, 3 months, and 6 months post-surgery. Gingival recession significantly reduced both in test and control groups with no intergroup difference. The exposed root was covered by 70% in the test group and 78% in the control group. Clinical attachment level, the width of keratinized tissue, recession height, and recession width was significantly increased in the case of coronally advanced flap alone with significant intragroup comparison. The results for both treatment techniques for recession coverage were compared. CAF displayed superior results than CAF along with Biomesh^®^ membrane in terms of clinical attachment level, root coverage percentage, and attached gingiva width.

## INTRODUCTION

In recent years, periodontal therapy, like dental therapy, has become increasingly focused on aesthetic outcomes that go beyond tooth replacement and color to include the soft tissue component [1]. Over the years, numerous surgical procedures have been used to correct gingival recession defects [2], and many multifold root coverage techniques have been advocated. Among them, coronally advanced flap (CAF) has been successful in treating type 1 (RT_1_) and type 2 (RT_2_) gingival recessions defects having a high rate of success [3], as this treatment can achieve optimal root coverage, effective color mixing of the treated area with neighboring tissues, and complete restitution of original soft tissue shape. Pedicle flaps have a less difficult postoperative healing than free gingival or connective tissue grafts.

More recently, researchers suggested the usage of guided tissue regeneration (GTR) techniques to re-establish soft tissue dimensions in the areas where they were lost. GTR efficacy and predictability in root coverage procedures were analyzed in various studies [4–7]. Membranes derived from poly (glycolic acid)(PLA), Poly (lactic acid) (PGA), (PLA/PGA) copolymers or collagen have different physical properties, and they are engrossed through different biologic processes, *i.e*., primarily through hydrolysis in the case of PLA/PGA copolymers and enzymatic degradation in case of collagen [8]. On the other hand, poly lactic-co-glycolic acid, or PLGA, is one of the most successful as it grants single-step procedures, thus reducing the patient inconvenience and potential surgical complexity [9].

The aim of this study was to evaluate and compare coronally advanced flap with and without (Biomesh^®^) with respect to root coverage, the width of keratinized gingiva, and clinical attachment level.

## MATERIAL AND METHODS

A randomized controlled study was carried out in the Department of Periodontology and Oral Implantology, Swami Vivekanand Subharti Dental College, Hospital, Meerut (Uttar Pradesh), India. All participants were recruited in this study within 1 month of getting clearance from the Ethical Committee.

### Study population

The study included 20 systemically healthy patients concerned about receding gums and unaesthetic smiles. An overly large sample size would cause more inconvenience to patients as per the objectives of the study. The patients with bilateral gingival recession were allotted arbitrarily into two groups: the control group, the coronally advanced flap alone was used to cover the recession defect, and the test group, the coronally advanced flap with (Biomesh^®^) membrane was used to cover up the recession defect.

Inclusion criteria were: 1) patient aged 20–40 years, 2) absence of medical history, periodontal surgery, and no prescribed drugs which interfere with periodontal tissue health or their healing in the forthcoming 6 months, 3) no underlying periodontal disease, 4) displaying recession type 1 (RT_1_) and type 2 (RT_2_), 5) probing depth of <3 mm, 6) width of keratinized gingiva >2 mm, 7) absence of bleeding on probing, 8) vital tooth and, 9) no caries and restorations.

Exclusion criteria were: 1) patients with underlying systemic diseases, untreated periodontal disease, 2) recession type 3 (RT3), 3) pregnant and lactating patients, 4) smokers, 5) uncooperative towards oral hygiene maintenance.

### Clinical Parameters

Clinical parameters were recorded in the test and control groups at different time intervals (baseline, 1, 3, and 6 months). Parameters were recorded amid-facial area, keeping CEJ as an anatomic reference point. Gingival recession was measured with the help of the UNC-15 probe and Vernier caliper.

1. Plaque index (PI): based on criteria by Silness and Loe [10];
2. Gingival index (GI): based on criteria by Loe and Silness [11];
3. Recession height (RH) (Figure 1 AB, Figure 2 AB);
4. Recession width (RW) (Figure 1 CD, Figure 2 CD);
5. Width of keratinized gingiva (WKG) (Figure 1 E, Figure 2 E);
6. Pocket probing depth (PPD);
7. Clinical attachment level (CAL);
8. Root coverage percentage.

**Figure 1AB:**
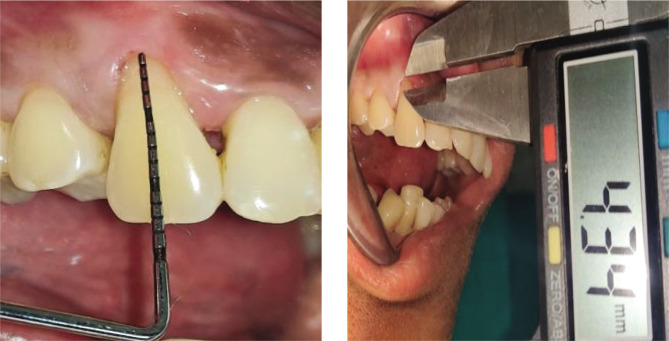
Pre-operative recession. Height using UNC-15 probe and Vernier caliper.

**Figure 2AB:**
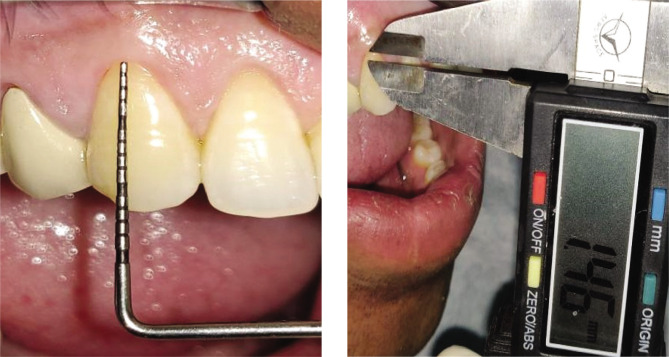
Pre-operative recession height using UNC-15 probe and Vernier caliper.

**Figure 1CD:**
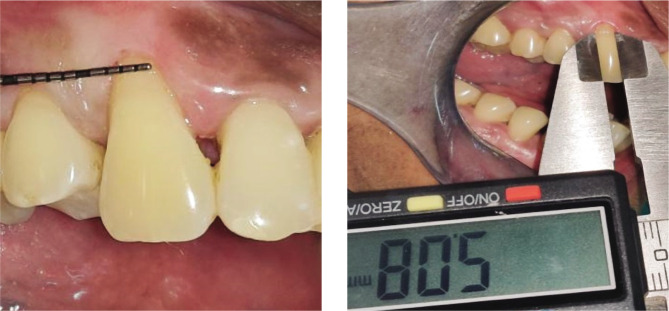
Pre-operative recession. Width using UNC-15 probe and Vernier caliper.

**Figure 2CD:**
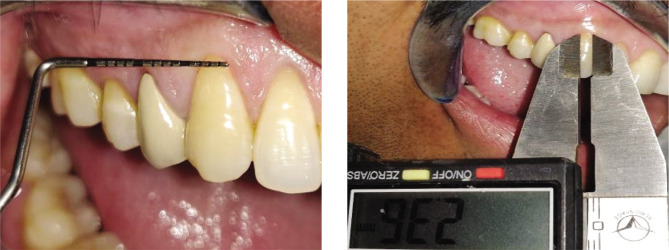
Pre-operative recession width using UNC-15 probe and Vernier caliper.

**Figure 1E:**
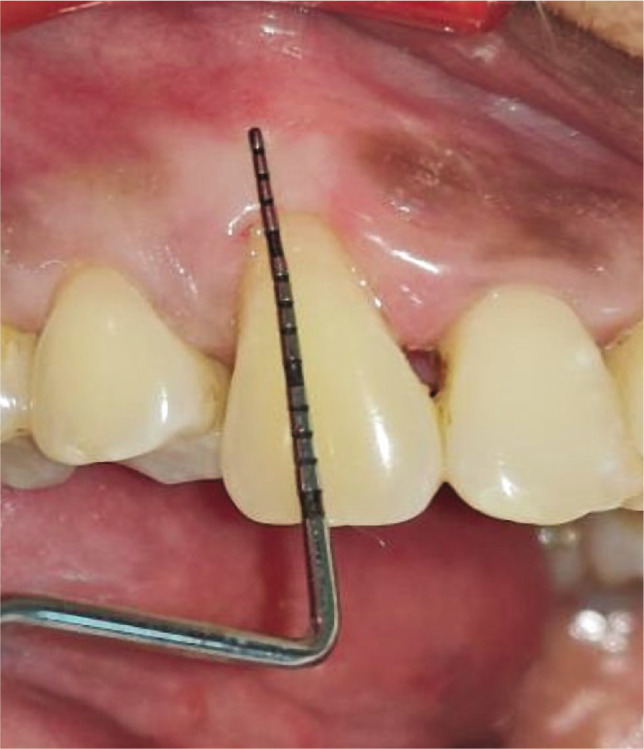
Pre-operative width of keratinized gingiva using UNC-15 probe.

**Figure 2E:**
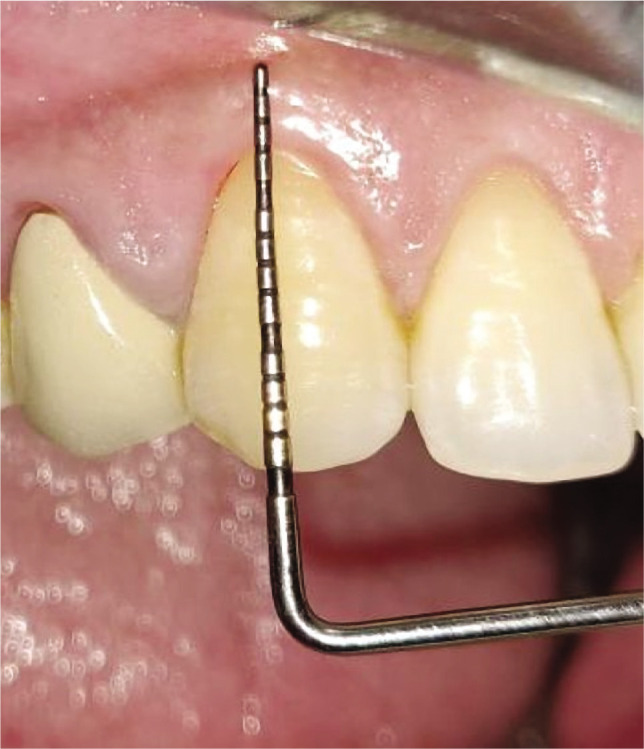
Pre-operative width of keratinized gingiva using UNC-15 probe.

All clinical parameters were recorded at baseline, 1 month, 3 months, and 6 months post-surgery. The periodontal treatment was performed by a single operator to avoid any bias in the study. The patient was enrolled in an oral hygiene program which included phase I therapy and oral hygiene instructions.

## SURGICAL PROCEDURE

### Control group

Adequate anesthesia was obtained at the surgical site with 2% lignocaine hydrochloride containing 1:2,00,000 epinephrine both at the recipient and donor site. The gingival recession was measured from CEJ to soft tissue margin (Figure 1 F, Figure 2 F). This vertical measurement was then applied at the mesial and distal interdental papilla to give two horizontal incisions on both sides of the tooth. Just above the cementoenamel junction (CEJ), a mesial and distal split-thickness horizontal incision was given on both sides of the tooth. From these horizontal incisions, two vertical incisions were given in the apical direction till alveolar mucosa (Figures 1 G, Figure 2 G). This whole trapezoidal design split-thickness flap was then elevated (leaving periosteum below to give blood supply to the overlying CAF) except at the crest of the marginal bone, where a full-thickness flap was elevated till the alveolar mucosa to maintain the maximal thickness of the tissue (Figure 1 H, Figure 2 H). Approximately 3 mm apical to the bone dehiscence at the apical portion of the flap at the lining mucosa of the lip split-thickness was performed again to make the flap accessible to coronally advanced with no tension. The most coronal aspect of the anatomic papilla was deepithelized to create the connective tissue bed for the anchorage of the coronally advanced flap. Root planing was performed with Gracey curettes.

**Figure 1F:**
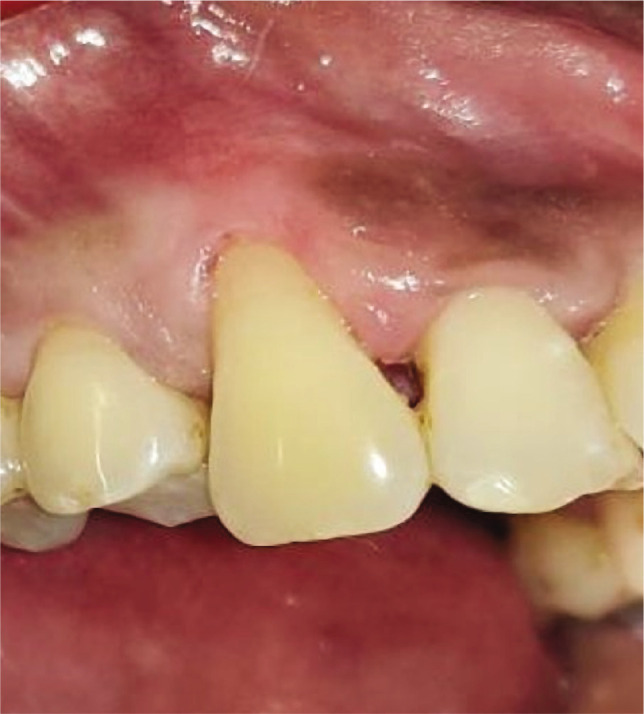
Pre-operative recession sitte #13.

**Figure 2F:**
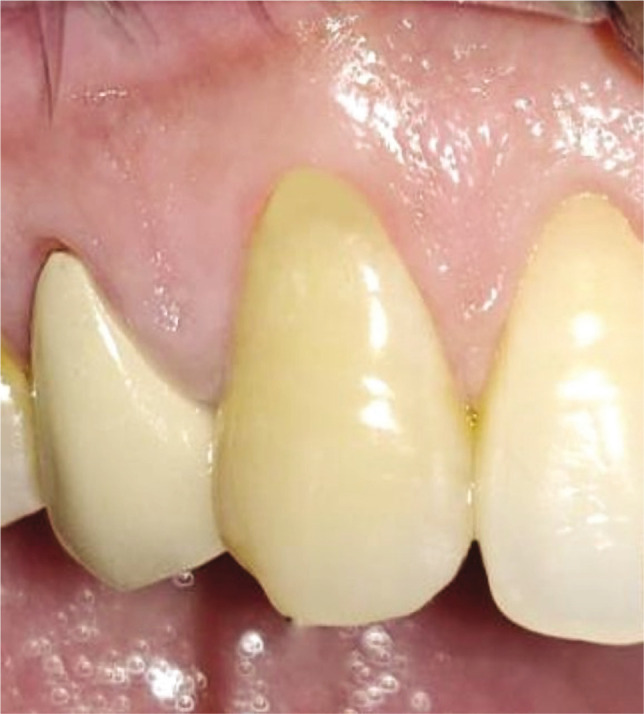
Pre-operative recession site #13.

**Figure 1G:**
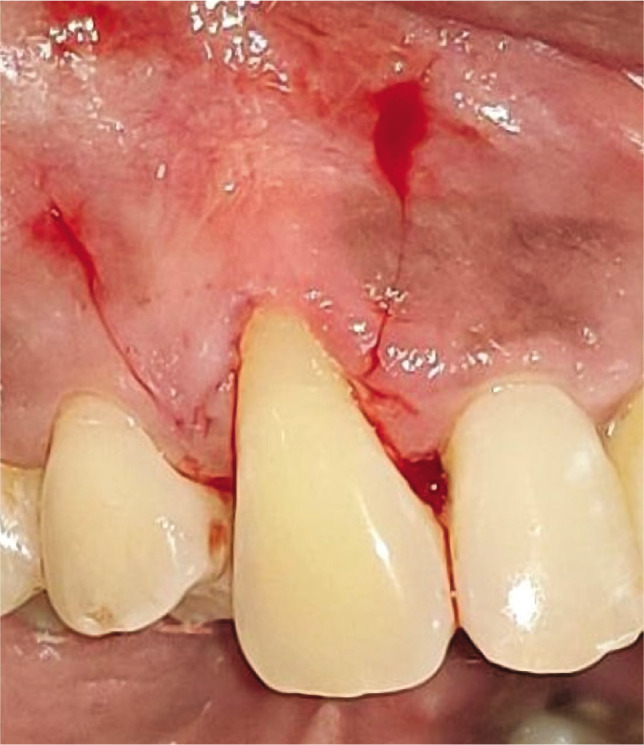
Vertical and horizontal incisions

**Figure 2G:**
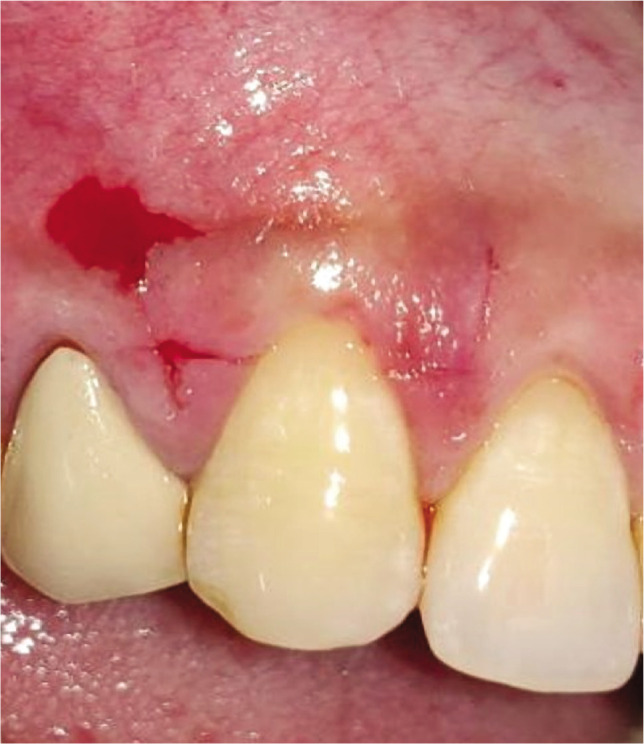
Vertical and horizontal incisions.

**Figure 1H:**
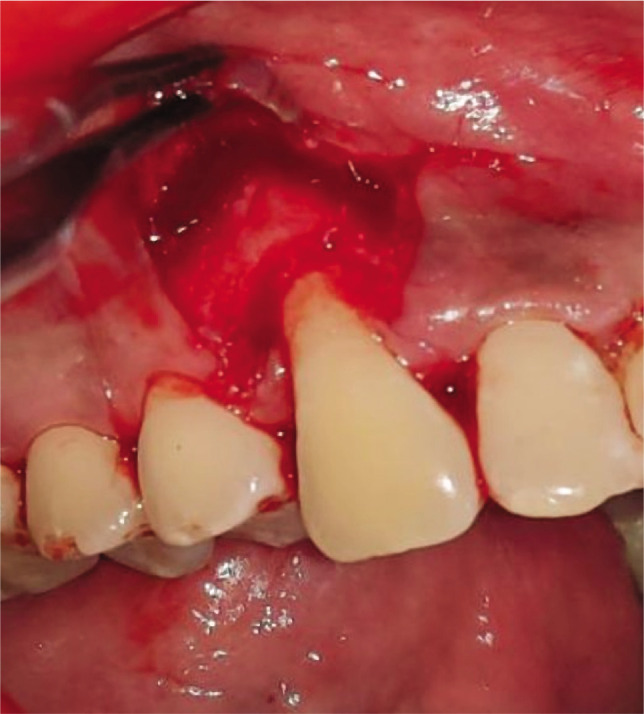
Split thickness flap reflected.

**Figure 2H:**
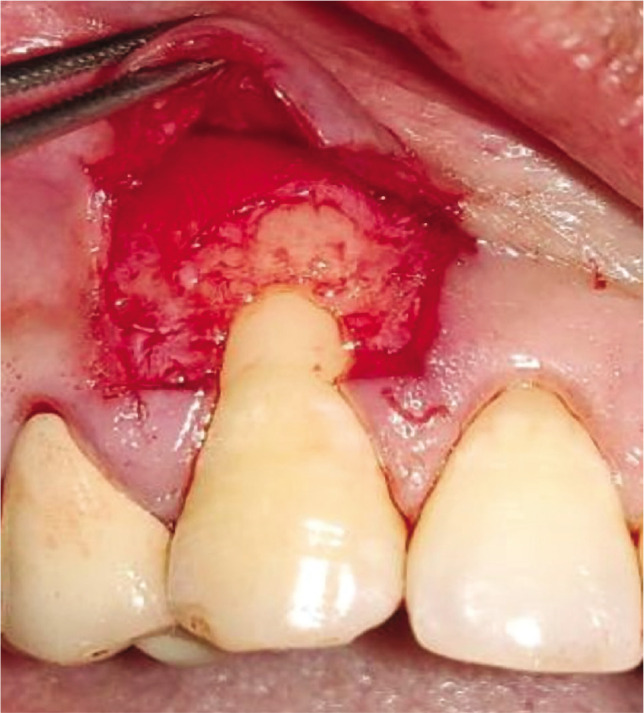
Split thickness flap reflected.

The CAF was then secured above the level of the cementoenamel junction at the base of the anatomic de-epithelized papilla. The vertical incisions were secured laterally by simple interrupted sutures and sling sutures in the papilla region using 5-0 polyglactin-910 (Figure 1 I, J). No postoperative complications were reported in any of the patients (Figure 1 K).

**Figure 1I:**
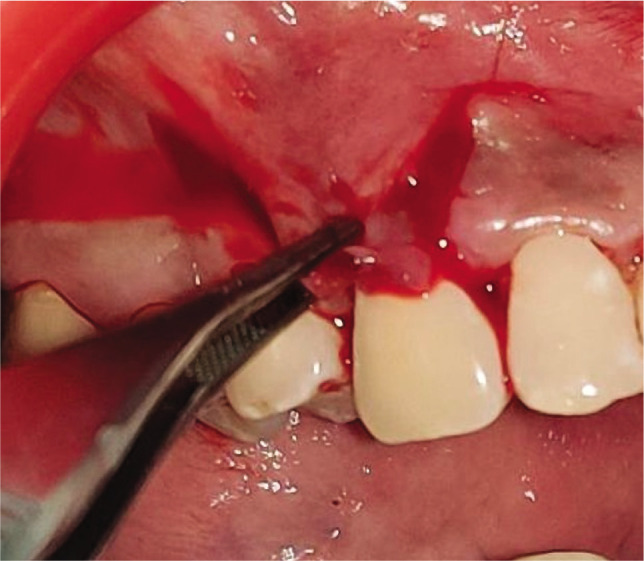
Corronally advanced flap.

**Figure 1J:**
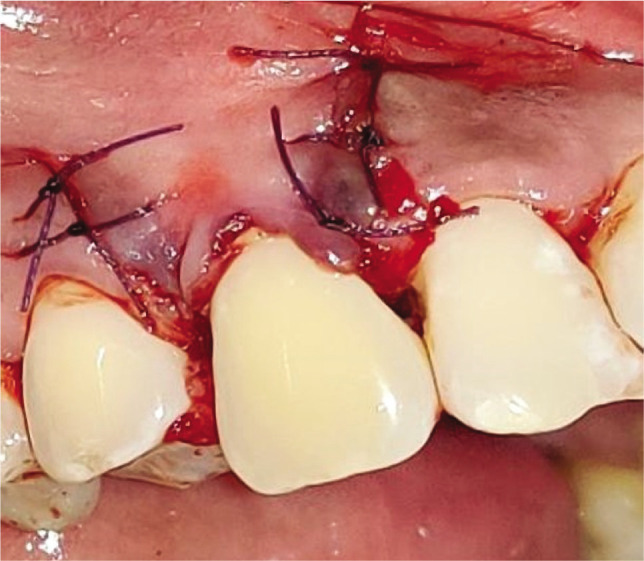
Sutures.

**Figure 1K:**
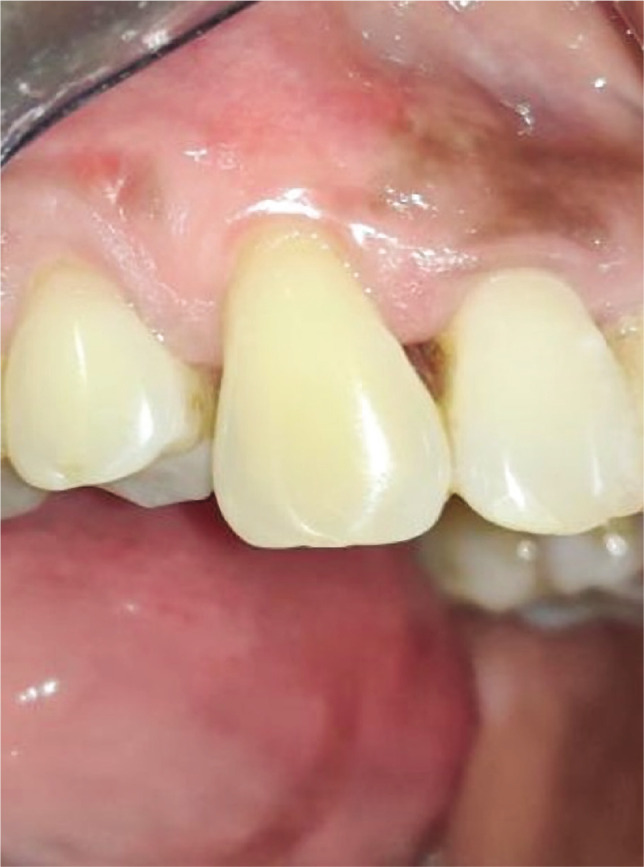
6-months post-operative.

### Test group

The CAF was prepared similarly to the control group. After recipient site preparation, a surgical template was trimmed as per the dimensions of the recipient site. After the surgical template was finalized, the Biomesh^®^ membrane was adapted and sutured around the recipient’s teeth at the level of CEJ. The membrane was sutured (Figure 2 I, J). The flap was advanced coronally to fully cover the membrane and sealed laterally with simple interrupted sutures and sling sutures at the interdental papilla to achieve a perfect fit around the teeth (Figure 2 K). Healing was uneventful, and no postoperative complications were reported (Figure 2 L).

**Figure 2I:**
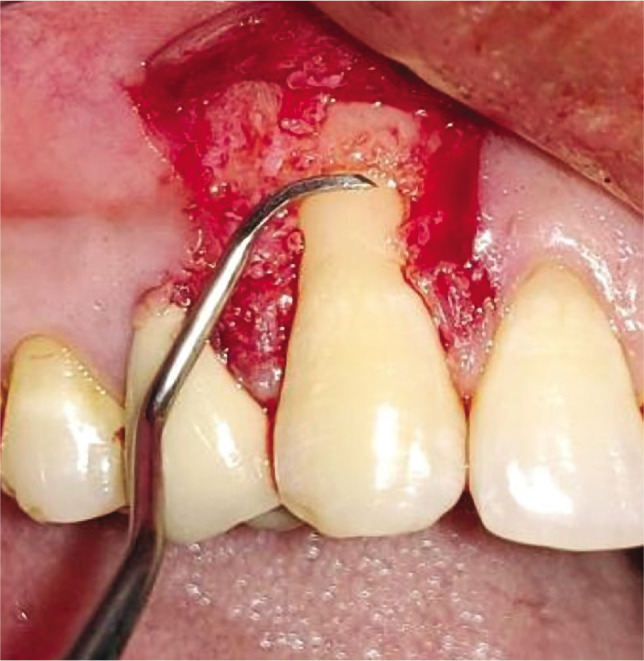
Root planing done.

**Figure 2J:**
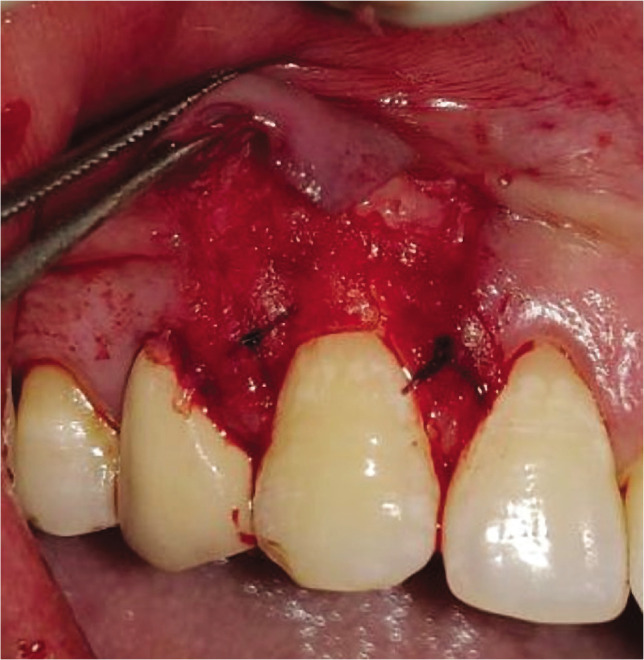
BIOMESH® membrane placed and sutured.

**Figure 2K:**
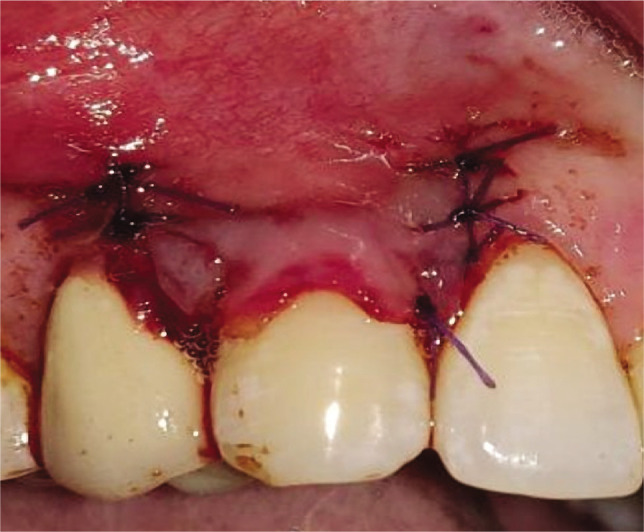
Coronally advanced flap sutured.

**Figure 2L:**
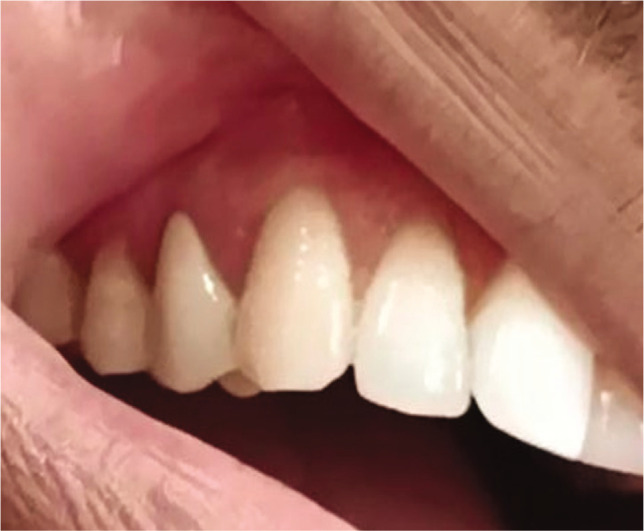
6-months post-operative.

### Post-surgical instructions

Patients were administered Amoxicillin plus Clavulanic acid 625 mg thrice daily for 5 days and Ibuprofen thrice daily for 3 days postoperatively. Patients were instructed to use Chlorhexidine (0.2%) mouth rinse for 30 seconds twice daily and avoid aggressive rinsing for the first 4 weeks. Patients were informed to report to the department in case of any discomfort. Patients were also instructed not to brush the operated area for the first 2 weeks and avoid disruptive food for the first month following the surgery. The sutures were removed after 15 days. After suture removal, all patients were instructed to initiate gentle brushing on the operating side with a soft bristle brush. All the patients were periodically recalled for checkups and clinical parameters recordings at 1, 3, and 6 months postoperatively.

## RESULTS

**Figure 3:**
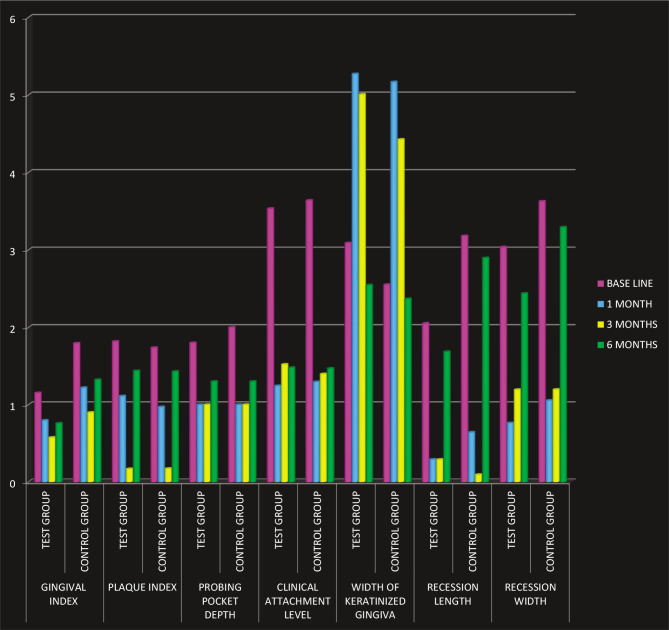
Average scores of different parameters among test and control groups measured at different time points.

**Figure 4:**
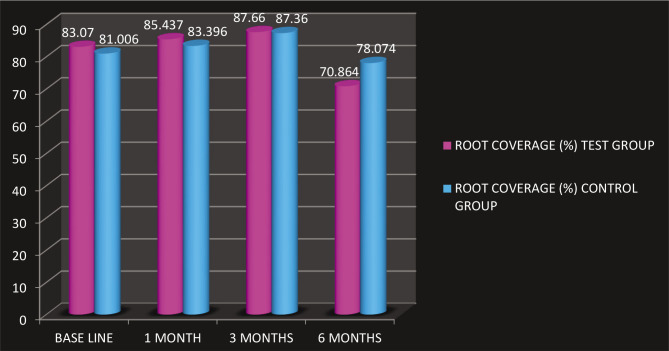
Average scores of root coverage in test and control groups at different time points.

**Figure 5:**
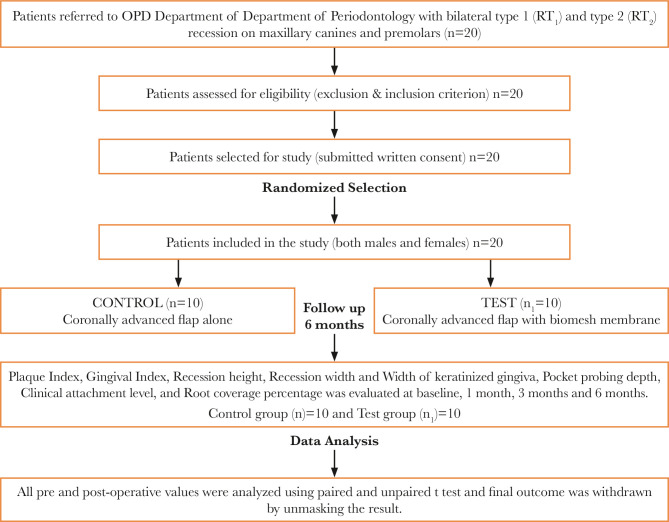
Consort Flow Chart.

All the base parameters are tabulated in (Table 1 and Figure 3, Table 2 and Figure 4). The mean full-mouth gingival and plaque scores for control and test at baseline were 1.79 0.15, 1.73±0.145 and 1.15 0.39, 1.73 0.14, At 6 months gingival scores reduced to 1.32 0.22, 1.81 0.11 and 0.75 0.33, 1.43 0.31 respectively. The plaque and gingival index showed significant differences from baseline to 6 months post-therapy. However, as this was a split-mouth study, these parameters did not differentiate between the test and the control groups. All patients tolerated the surgical procedures well, experienced no postoperative complications, and complied with the study protocol. The reduction in GH was significant in both the test and control groups (p<0.05), with no differences between groups. The exposed root was covered by 70.8% 2.85% in the test group and 78.0% 6.04% in the control group. Significant CAL gain and gain in WKG were observed in the case of CAF on intragroup comparison and were measured by adding the depth of the pocket and the recession height. The decrease in PPD showed no difference between groups. The mean RW score for the test group at 6 months was 2.430.88 mm and for the control group was 3.29±1.12 mm. The difference between the scores was not statistically significant. Healing was uneventful, and no postoperative complications were reported in any of the patients. No patient was dropped out or excluded from the study, as seen in Figure 5.

**Table 1:** The probable values of paired t-test within successive time intervals in control group and test group for all parameters (Intra group/within group comparison).

S.NO.	Parameters	Control				Test				
		0–1	1–3	3–6	% Improvment	0–1	1–3	3–6	% Improvment	
**1**	Gingival index	.0965** P>.05 (N.S.)	.1025** P>.05 (N.S.)	.0996** P>.05 (N.S.)	73.74%	.0954** P>.05 (N.S.)	.0854** P>.05 (N.S.)	.0942** P>.05 (N.S.)	65.86%	
**2**	Plaque index	.1124** P>.05 (N.S.)	.1020** P>.05 (N.S.)	.0987** P>.05 (N.S.)	82.22%	.0889** P>.05 (N.S.)	.0941** P>.05 (N.S.)	.0654** P>.05 (N.S.)	78.66%	
**3**	Probing pocket depth	.0985** P>.05 (N.S.)	1** P>.05 (N.S.)	1** P>.05 (N.S.)	65.00%	.0883** P>.05 (N.S.)	1** P>.05 (N.S.)	1** P>.05 (N.S.)	72.22%	
**4**	Clinical attachment level	.0000* P<.05 (SIG.)	.0041* P<.05 (SIG.)	.0048* P<.05 (SIG.)	43.85%	.0000* P<.05 (SIG.)	.0235* P<.05 (SIG.)	.0052** P<.05 (SIG.)	40.40%	
**5**	Width of keratinized gingiva	.0000* P<.05 (SIG.)	.0190* P<.05 (SIG.)	.0016* P<.05 (SIG.)	92.90%	.0000* P<.05 (SIG.)	.0273* P<.05 (SIG.)	.0018* P<.05 (SIG.)	82.42%	
**6**	Recession height	.0000* P<.05 (SIG.)	.0042* P<.05 (SIG.)	.0257* P<.05 (SIG.)	91.10%	.0000* P<.05 (SIG.)	.0049** P<.05 (SIG.)	.0023** P<.05 (SIG.)	82.16%
**7**	Recession width	.0000* P<.05 (SIG.)	.0034** P<.05 (SIG.)	.0024* P<.05 (SIG.)	90.79%	.0000* P<.05 (SIG.)	.0135* P<.05 (SIG.)	.0507* P<.05 (SIG.)	80.28%
**8**	Root coverage (%)	.0003* P<.05 (SIG.)	.0038* P<.05 (SIG.)	.0042* P<.05 (SIG.)	96.28%	.0012* P<.05 (SIG.)	.0135* P<.05 (SIG.)	.0507* P<.05 (SIG.)	85.30%

* – Significant difference between different time intervals at .05 level of significance <.05. ** – No significant difference between different time intervals at .05 level of significance p>.05

**Table 2:** Comparison of mean & standard deviation of different parameters in test & control group (independent t-test). Inter group/between group comparison.

S.NO.	Parameters	Time-points	Mean±S.D.		t-value (calculated) & (crit.)=1.96	P-values/Significance
			Test group	Control group		
**1**	Gingival index	Base line	1.151±.393	1.794±.157	.1056	.1305** P>.05 (N.S.)
		1 month	.797±.309	1.220±.293	.0332	.0856** P>.05 (N.S.)
		3 months	.575±.337	.899±.351	.1887	.0693** P>.05 (N.S.)
		6 months	.758±.336	1.323±.228	.1669	1081**P>.05 (N.S.)
**2**	Plaque index	Base line	1.818±.113	1.738±.145	.2114	.1862** P>.05 (N.S.)
		1 month	1.112±.237	.971±.104	.3551	.1104** P>.05 (N.S.)
		3 months	.169±.036	.172±.117	.0889	.9398** P>.05 (N.S.)
		6 months	1.438±.316	1.429±.317	.1132	.1175** P>.05 (N.S.)
**3**	Probing pocket depth	Base line	1.8±.789	2.816	.4552	.5843** P>.05 (N.S.)
		1 months	1±0	1±0	.0000	1** P>.05 (N.S.)
		3 months	1±0	1±0	.0000	1** P>.05 (N.S.)
		6 months	1.3±.675	1.3±.483	.8891	1** P>.05 (N.S.)
**4**	Clinical attachment level	Base line	3.532±.652	3.639±.489	.4555	.9449** P>.05 (N.S.)
		1 month	1.244±.310	1.296±.371	.7741	.7378** P>.05 (N.S.)
		3 months	1.522±.289	1.3.98±.244	.2697	.3131** P>.05 (N.S.)
		6 months	1.478±.359	1.470±.305	.6523	.9578** P>.05 (N.S.)
**5**	Width of keratinized gingiva	Base line	3.089±.322	2.551±.198	.5446	1** P>.05 (N.S.)
		1 month	5.271±.373	5.167±.421	.5889	.5658** P>.05 (N.S.)
		3 months	5.007±.477	4.426±.940	.9981	.3259** P>.05 (N.S.)
		6 months	2.546±.800	2.370±.256	.1996	.6478** P>.05 (N.S.)
**6**	Recession height	Base line	2.052±.845	3.180±.613	.3336	.1134** P>.05 (N.S.)
		1 month	.291±.550	.642±.689	.1556	.2250** P>.05 (N.S.)
		3 months	.290±.468	.094±.052	.7448	.2197** P>.05 (N.S.)
		6 months	1.686±.868	2.897±.345	.7789	.0375** P>.05 (N.S.)
**7**	Recession width	Base line	3.038±1.104	3.627±1.007	.6993	.1841** P>.05 (N.S.)
		1 month	.763±.292	1.055±1.034	.8003	.4096** P>.05 (N.S.)
		3 months	1.195±.478	1.197±.554	.7945	.9932** P>.05 (N.S.)
		6 months	2.439±.887	3.2931.128	.9912	.3878** P>.05 (N.S.)
**8**	Root coverage (%)	Base line	83.070±2.024	81.006±3.877	.8725	.1552** P>.05 (N.S.)
		1 month	85.437±2.081	83.396±3.983	.5542	.4096** P>.05 (N.S.)
		3 months	87.660±.850	87.360±.605	.1996	.9932** P>.05 (N.S.)
		6 months	70.864±2.852	78.074±6.044	.6693	.3878** P>.05 (N.S.)

* – Significant difference between different time intervals at .05 level of significance p<.05. ** – No significant difference between different time intervals at .05 level of significance p>.05.

## DISCUSSION

This randomized, controlled, split-mouth clinical study evaluated root coverage by comparing coronally advanced flap with and without Biomesh^®^ membrane in type 1 (RT_1_) and type 2 (RT2) recession defects. The CAF design described by de Sanctis and Zucchelli comes with distinctive assets in terms of clinical and biological considerations compared to traditional techniques mentioned by Allen and Miller [12]. In a study, CAF with releasing incisions was compared to an envelope flap without releasing incisions, and it was concluded that the releasing incisions resulted in a significantly greater percentage of defect coverage (95%) than the procedure without releasing incisions (78%) [13, 14]. A split-full-split-thickness flap design is an important factor for wound healing and thus the accomplishment of complete root coverage. The periosteum is considered ‘UMBILICAL CORD’ as it is rich in vascular plexus [15]. It has boundless regenerative potential due to various cells and growth factors like vascular endothelial growth factor, which boosts revascularization in wound healing.

The presence of thick gingiva is beneficial as it charters more blood vessels and relaxes surgical manipulation [16]. The placement of the gingival margin was 2 mm above CEJ to counterbalance gingival deflation during the healing phase following surgery [17]. Both sites were treated with CAF, and healing was uneventful with respect to gingival color, texture, and contour resembling adjacent soft tissue.

Guided tissue regeneration (GTR) is based on guiding the proliferation of various cells during healing following periodontal surgery. The potential advantage of GTR is the possibility of achieving periodontal regeneration rather than connective tissue repair to the exposed root surfaces. The technique of using barriers was introduced by Nyman et al. [18], and the term GTR was coined by Gotlow et al. [19], which works upon the principle of selective cell repopulation or controlled tissue regeneration using periodontal ligament cells [20].

Biomesh^®^ (developed by Samyang Corporation^®^, South Korea) is a microporous membrane made of biodegradable polyglycolic-polylactic acid copolymer [21]. It posed assertive drawbacks during handling, especially adaptation at the surgical site. Because of stiffness, there were increased chances (after suture removal) that the membrane might rebound and may open the incision line and delay the healing process [22]. However, this limitation can be overcome by adding softeners like N-methyl- 2-pyrrolidone (NMP). NMP has been shown to soften PLGA membranes and speed up preosteoblastic cell maturation and bone regeneration in recent studies. While PLA and PLGA-based membranes are non-cytotoxic and biodegradable, oligomers and acid byproducts released during degradation can cause inflammation and a foreign body response in vivo, and because of this, some of our patients complained of postoperative pain at the membrane site. However, healing remained uneventful [23].

## CONCLUSION

The results for both treatment techniques for recession coverage were compared. CAF displayed superior results than CAF along with Biomesh^®^ membrane in terms of clinical attachment level, root coverage percentage, and attached gingiva width. The limitation of the present study is the usage of the synthetic membrane, which may cause difficulty during handling and after suture removal as there are chances of membrane exposure. In addition, the study has a short follow-up period, and it should be compared with other treatment modalities.

## References

[1] (1992). Glossary of periodontal terms. http://www.worldcat.org/oclc/28019554.

[2] Langer B, Langer L (1985). Subepithelial connective tissue graft technique for root coverage. J. Periodontol.

[3] Shkreta M, Stojanovska A, Dollaku B, Belazelkoska Z (2018). Exploring the Gingival Recession Surgical Treatment Modalities: A Literature Review. Maced J Med Sci.

[4] Danesh-Meyer MJ, Wikesjo UM (2001). Gingival recession defects and guided tissue regeneration: a review. J. Periodontal Res.

[5] Tinti C, Vincenzi G, Cortellini P, Pini Prato G, Clauser C (1992). Guided tissue regeneration in the treatment of human facial recession. A 12-case report. J Periodontol.

[6] Harris RJ (1997). A comparative study of root coverage was obtained with guided tissue regeneration utilizing a bioabsorbable membrane *versus* the connective tissue with partial-thickness double pedicle graft. J. Periodontol.

[7] Trombelli L, Scabbia A, Tatakis DN, Calura G (1998). Subpedicle connective tissue graft *versus* guided tissue regeneration with the bioabsorbable membrane in the treatment of human gingival recession defects. J. Periodontol.

[8] Stavropoulos A, Sculean A (2004). GTR treatment of intrabony defects with PLA/PGA copolymer or collagen bioresorbable membranes in combination with deproteinized bovine bone (BioOss^®^). Clin Oral Investigations.

[9] Terriza A, Perez JA, Orden E, Yubero F (2014). Osteoconductive potential of barrier NanoSiO2 PLGA membranes functionalized by plasma-enhanced chemical vapor deposition. BioMed Res. Int.

[10] Silness J, Loe H (1964). Periodontal disease in pregnancy II. Correlation between oral hygiene and periodontal conditions. Acta Odontol Scand.

[11] Loe H, Silness J (1963). Periodontal disease in pregnancy I. Prevalence and severity. Acta Odontol Scand.

[12] Miller PD (1987). Root coverage with the free gingival graft. Factors associated with incomplete coverage. J Periodontol.

[13] Felipe Grisi (2007). Comparison of Two Surgical Procedures for Use of the Acellular Dermal Matrix Graft in the Treatment of Gingival Recessions: A Randomized Controlled Clinical Study. J Periodontol.

[14] Papageorgakopoulos G, Greenwell H, Hill M, Vidal R, Scheetz JP (2008). Root Coverage Using Acellular Dermal Matrix and Comparing a Coronally Positioned Tunnel to a Coronally Positioned Flap Approach. J. Periodontol.

[15] Chanavaz M (1995). The periosteum: the umbilical cord of bone quantification of the blood supply of cortical bone of periosteal origin. Rev Stomatol Chir Maxillofac.

[16] Hwang D, HL Wang (2006). Flap thickness as a predictor of root coverage: A systemic review. J Periodontol.

[17] Pini-Prato G, Carlo B, Nieri M, Franseschi D (2005). Coronally advanced flap: the post-surgical position of the gingival margin is an important factor in achieving complete root coverage. J Periodontol.

[18] Gottlow J, Nyman S, Karring T (1990). Guided Tissue Regeneration following treatment of recession type defects in the monkey. J. Periodontol.

[19] Gottlow J, Nyman S, Karring T, Wennstrom J (1986). New attachment formation in the human periodontium by guided tissue regeneration. Case reports. J Clin Periodontol.

[20] Hardwick R, Hayes BK, Flynn C (1995). Devices for dentoalveolar regeneration: an up to date literature review. J Periodontol.

[21] Mopur JM, Devi TR, Ali SM, Srinivasa TS (2013). Clinical and Radiographic Evaluation of Regenerative Potential of GTR membrane (Biomesh^®^) along with Alloplastic bone graft (Biograft^®^) in the treatment of periodontal intrabony defects. J Contemp Dent Pract.

[22] Wang J, Wang L, Zhou Z, Lai H (2016). Biodegradable Polymer Membranes Applied in Guided Bone/Tissue Regeneration: A Review. Polymers Basel.

[23] Gottlow J (1993). Guided Tissue Regeneration Using Bioresorbable and Non-Resorbable Devices: Initial Healing and Long-Term Results. J. Periodontol.

